# Individual recognition and long-term memory of inanimate interactive agents and humans in dogs

**DOI:** 10.1007/s10071-022-01624-6

**Published:** 2022-05-05

**Authors:** Judit Abdai, Dalma Bartus, Sylvain Kraus, Zsuzsanna Gedai, Beatrix Laczi, Ádám Miklósi

**Affiliations:** 1 MTA-ELTE Comparative Ethology Research Group, Budapest, Hungary; 2 University of Veterinary Medicine, Budapest, Hungary; 3 Paris 13 University, Paris, France; 4 Department of Ethology, Eötvös Loránd University, Budapest, Hungary

**Keywords:** Individual recognition, Memory, Animal-robot interaction, Robot, Dog

## Abstract

Investigation of individual recognition (IR) is difficult due to the lack of proper control of cues and previous experiences of subjects. Utilization of artificial agents (Unidentified Moving Objects: UMOs) may offer a better approach than using conspecifics or humans as partners. In Experiment 1, we investigated whether dogs are able to develop IR of UMOs (that is stable for at least 24 h) or that they only retain a more generalised memory about them. The UMO helped dogs to obtain an unreachable ball and played with them. One day, one week or one month later, we tested whether dogs display specific behaviour toward the familiar UMO over unfamiliar ones (four-way choice test). Dogs were also re-tested in the same helping context and playing interaction. Subjects did not approach the familiar UMO sooner than the others; however, they gazed at the familiar UMO earlier during re-testing of the problem solving task, irrespectively of the delay. In Experiment 2, we repeated the same procedure with human partners, applying a two-way choice test after a week delay, to study whether lack of IR was specific to the UMO. Dogs did not approach the familiar human sooner than the unfamiliar, but they gazed at the familiar partner earlier during re-testing. Thus, dogs do not seem to recognise an individual UMO or human after a short experience, but they remember the interaction with the novel partner in general, even after a long delay. We suggest that dogs need more experience with a specific social partner for the development of long-term memory.

**Supplementary Information:**

The online version contains supplementary material available at 10.1007/s10071-022-01624-6.

## Introduction

The ability to recognize others individually is advantageous for social animals. Individual recognition (IR) involves (1) individually distinctive cues displayed by the subject that can be (2) learned by the observer. These cues (3) allow matching current sensory input with the previously learned features in future interactions, and (4) form the basis of showing specific behaviour toward others based on their identity (e.g. Gherardi et al. 2010; Proops et al. 2009; Tibbetts and Dale 2007; see also the review by Gherardi et al. 2012). IR is thought to be widespread among animals due to its advantages in several social contexts, such as mate or kin recognition, or in dominance hierarchies (Dale et al. 2001). IR can be based on unique visual, acoustic or olfactory features that allow distinction between individuals. Potentially, IR could have evolved in any species where repeated interaction among group members is likely to occur frequently or the cost of competition can be reduced by showing individual-specific behaviour toward rivals (e.g. Aubin et al. 2000; Carazo et al. 2008; Madeira and Oliveira 2017; Sheehan and Tibbetts 2009).

In contrast to IR, class-level recognition (CLR) is based on characteristics shared by many individuals in the group, who may represent certain subgroups of sex, age or hierarchical rank (Gheusi et al. 1994; Madeira and Oliveira 2017). CLR and IR diverge in important ways, but many studies do not aim to discriminate between the two or are missing required controls to disentangle these mental skills (see Gábor et al. 2019). It is difficult to properly control for all cues displayed or emitted by the partners, and to limit the role of subjects’ previous experience, both of which are important to distinguish IR from CLR. Application of robots may facilitate the investigation of IR, because this allows researchers to gradually change the morphological and behavioural features of the partner, and depending on its embodiment (similarity to known social partners) the influence of previous experiences can be limited as well (Abdai et al. 2018; Frohnwieser et al. 2016). Further advantages of deploying robots is that both the quality and the quantity of the (direct or third-party) experience with the social partner can be controlled, allowing researchers to study how these aspects contribute to IR.

Individual variability in vocalization is widespread in the Canidae family. For example, it has been shown in the howling of wolves (*Canis lupus*) (Palacios et al. 2007; Root-Gutteridge et al. 2014a, b; Tooze et al. 1990), and researchers also found individual-specific variation in dog (*Canis familiaris*) barks (Molnár et al. 2008; Yin and McCowan 2004). Although these acoustic differences might be used to identify others, the presence of individually distinctive cues does not necessarily indicate the functioning of IR (see Schibler and Manser 2007; Yorzinski 2017). Further, unique visual and olfactory cues may also contribute to the IR of conspecifics in dogs (and wolves). Hepper (1994) found that dogs show preference toward their mother/offspring (vs unfamiliar, unrelated individual), but not toward their siblings after a two-year separation, relying solely on olfactory cues. Hamilton and Vonk (2015) found that dogs are able to recognize kin without familiarity (although in females discrimination was not clear), and the study of Lisberg and Snowdon (2011) further showed that female dogs are able to discriminate between familiar and unfamiliar individuals based on olfactory cues alone. These findings show that dogs are able to rely on olfactory cues to discriminate among conspecifics; however, dogs might show CLR and not IR.

Identifying humans individually can be important for domestic dogs. Intraspecific IR in their ancestors might have favoured the emergence of heterospecific IR, but domestication and developmental experience could also contribute to the occurrence of this cognitive skill. Advantages of being able to discriminate between humans individually, and their social environment during development support the notion that dogs have the cognitive skill to identify humans based on individually distinctive cues.

In previous studies, dogs were able to find the owner based on olfactory cues alone when they were close to him/her; however, not when the owner and the two unfamiliar humans were three metres away from the dog (Polgár et al. 2015). Dogs could also locate their owner based on his/her voice alone, when the other choice was an unfamiliar person (Gábor et al. 2019). Although these studies show that dogs discriminate their owner from unfamiliar people based solely on olfactory and vocal cues, in both cases subjects could rely on the degree of familiarity (CLR). Regarding visual cues, dogs are able to discriminate between pictures of humans and dogs that they have seen before vs novel ones; however, discrimination could be the result of familiar vs unfamiliar cues here as well (Racca et al. 2010). Huber et al. (2013) found that dogs are able to discriminate between their owners and a familiar human relying solely on their heads (live presentation and picture). Although subjects had difficulties choosing their owner when only the inner part of their faces were displayed (picture). Furthermore, Adachi et al. (2007) reported that dogs are able to recall their owner’s face upon hearing their voice, thereby demonstrating cross-modal representation of their owner. Thus, it seems dogs are able to individually discriminate their owner.

Despite only having empirical data on the recognition of owners, who represent a specific category within humans, it is also assumed that dogs are able to discriminate other humans individually. However, we do not know the quality and quantity of (direct or third-party) social interaction that is required for dogs to be able to identify humans individually. We also do not know and the type of cues dogs rely on in doing so, and the duration for which dogs can remember to a specific individual.

Dogs display social behaviour toward an unfamiliar, self-propelled object (UMO—Unidentified Moving Object) (e.g. Abdai et al. 2015). Across studies, researchers manipulated whether the UMO was merely a moving object (moving around the room without engaging in interaction with the dog) or the UMO interacted shortly with the dog in a problem solving task, helping the dog to obtain an unreachable reward. Right after the short interaction, dogs learnt to follow the communicative signals of the UMO, but they failed to learn the indication of the UMO when it was presented as a moving object (Gergely et al. 2015). Interactive UMOs were also able to elicit social bias in dogs (Abdai et al. 2015; Gergely et al. 2016). When presenting dogs with a free choice between larger and smaller food quantity, the UMO indicated the option opposite to the dogs’ initial preference. We found that the interactive UMO was able to revert dogs’ choice for the small amount; however, the UMO’s indication had no effect on dogs’ choice when it did not show interactive behaviour before (Abdai et al. 2015). Gergely et al. (2016) also found that dogs commit the A-not-B error when the interactive UMO was hiding the ball, but the error did not occur when the partner was the non-interactive UMO. Based on these results, UMOs could be applied as partners to investigate IR in dogs, providing better control over the characteristics of the potential social partners.

Here our aim was to test the UMO’s utility to study IR and memory in dogs. In Experiment 1, we investigated whether dogs are able to recognize an UMO that they previously interacted with in two situations. Considering that this method has never been used before, for comparison, in Experiment 2 we applied a similar procedure to test dogs’ behaviour when human partners were presented.

## Experiment 1

We used UMOs as interactive partners in a problem solving and playful situation, and retested subjects one day, one week or one month after the short social interaction. We hypothesised that dogs would remember the individual UMO because it had helped them to solve a problem, and it had shown playful behaviour. Thus, dogs would be motivated to engage in interaction with this partner again. We predicted that dogs would show specific behaviour toward the familiar UMO after a day or a week, but they would be less likely to remember it after a month due to the short duration of the initial interaction. Considering that the UMOs were a novel social partner, we expected that even if dogs would not recognize the specific individual, they would remember the helping and playful behaviour of the UMO in general. In previous studies, dogs’ behaviour toward the UMO had been tested right after familiarization (Abdai et al. 2015; Gergely et al. 2015, 2016), but in the present experimental setup our aim was to investigate dogs’ memory after a longer delay.

During the first occasion, the UMO was helping subjects to obtain a ball from an unreachable location. The UMO reacted to the gazing behaviour of the dog, that is, it started to move during the problem-solving task when the dog looked at it. After obtaining the ball, the UMO also attempted to engage in a playing interaction with the dog. One day, one week or one month later (between-subject design), we tested whether dogs remembered the individual UMO, i.e. whether they showed preference toward the familiar UMO in a four-way choice test. Following this, dogs faced the same problem solving task and playful interaction as during their first encounter with the UMO. Here we applied a playing interaction instead of using food as motivation (cf. Abdai et al. 2015; Gergely et al. 2015), because we aimed to investigate whether dogs are able to remember the UMO based on the social experience, and not because it provided food to them.

### Methods

#### Subjects

We included dogs above one year of age that could be motivated to participate by a tennis ball based on the owner’s opinion. Out of 74 dogs, 27 had to be excluded for various reasons. We excluded eight dogs because they showed distress either in the room or in the presence of the UMO, three dogs because they continuously attacked the UMO, four dogs because they did not come to the retest, seven dogs because they were not motivated by the ball, and one dog because it did not give the ball back neither to the experimenters, nor to the owner. We further excluded four dogs due to procedural issues (e.g. the UMO failed to retrieve the ball from the cage repeatedly or the owner did not follow the instructions properly). Thus, 47 dogs remained in the final analyses (different breeds, 20 females; mean ± SD age: 4.5 ± 3.0 years). We assigned dogs to three groups based on the time passing between the first occasion and the retest, which depended on the availability of the owner: Day group (*N* = 15; 8 females, mean ± SD age: 4.4 ± 3.6 years), Week group (*N* = 16; 8 females; mean ± SD age: 5.2 ± 2.8 years), and Month group (*N* = 16; 4 females; mean ± SD age: 4.0 ± 2.5 years) (for more details see Online Resource 2).

#### Test partner and apparatus

**Fig. 1:**
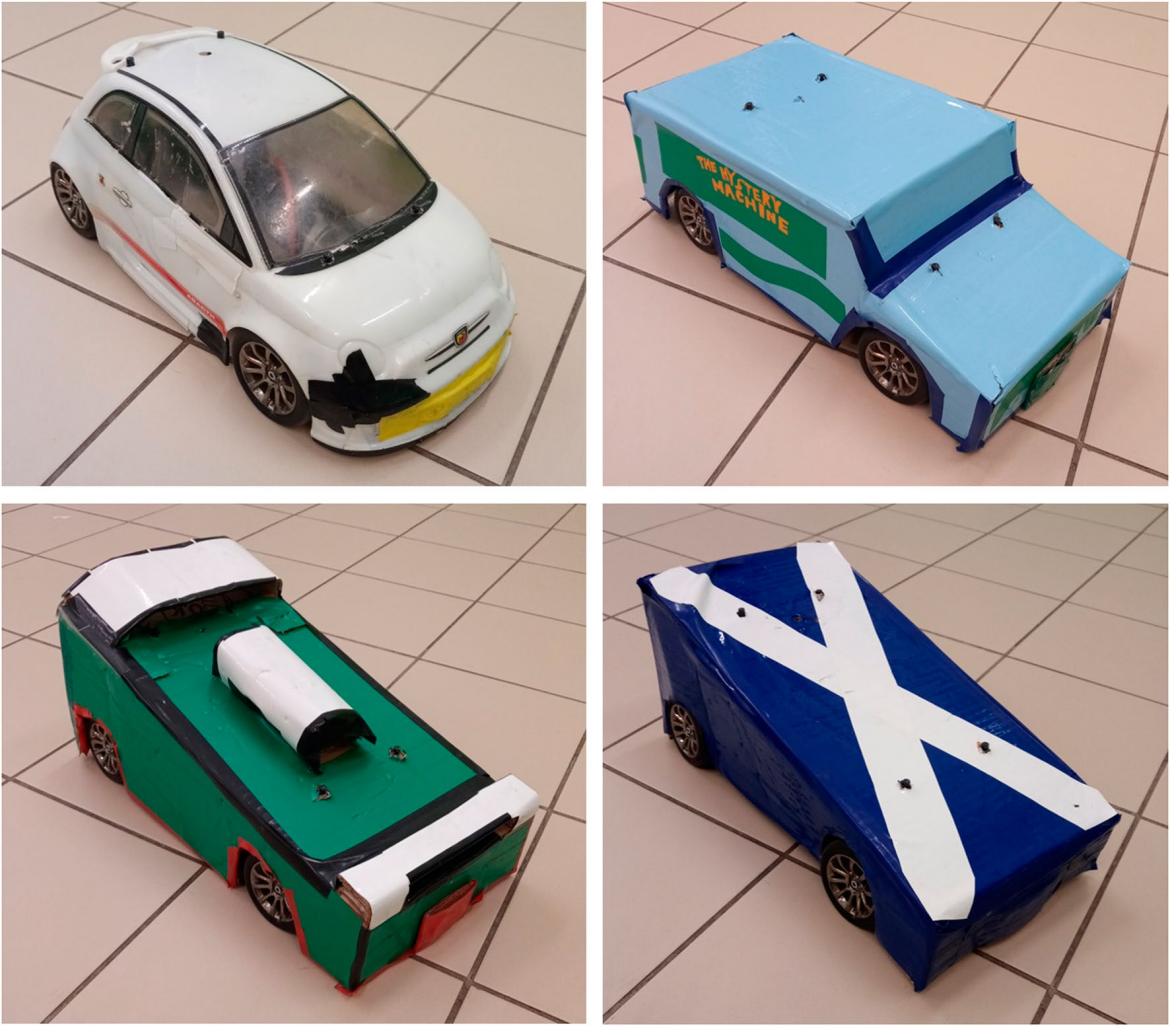
Embodiments of the UMOs

We used a remote controlled car (#32710 RTR Switch Abarth 500, basis: 28 cm × 16 cm × 13 cm) as an interactive partner (UMO) which could be covered with four different embodiments differing in colour and shape (Fig. 1). Three embodiments of the UMO were handcrafted from cardboard boxes and self-adhesive wallpapers, and the fourth was the original plastic cover of the remote controlled car. We counterbalanced within groups, which UMO was presented as familiar. The UMO was controlled by Experimenter (E) 1 from outside through two fish-eye optic cameras.

Dogs were tested in a 6.27 m × 5.40 m room at the Department of Ethology, Eötvös Loránd University, Budapest, Hungary. All tests were recorded with two fish-eye optic cameras (Mobius ActionCam) attached to the ceiling. We used a tennis ball to motivate dogs. During the problem solving task, we put the ball inside a wire-mesh cage (L × W × H: 61 cm × 47 cm × 54.5 cm) with a front opening (W × H: 20 cm × 18 cm). The ball was placed on a plastic plate (8 cm × 8 cm) with metal sheets on its sides, and the plate was attached to magnets on the bottom of the cage to prevent dogs from getting the ball by moving the cage. All embodiments of the UMO had magnets on their front to be able to attach to the plate and thus bring it out of the cage.

In the Test phase of the Recognition Session (see below), we used an occluder (125 cm × 100 cm with two bent sides of 125 cm × 70 cm) to cover the dog’s view of the room while E1 placed the four UMOs and the balls inside the room. For more details about the apparatus, see Online Resource 1.

#### Procedure

**Fig. 2:**
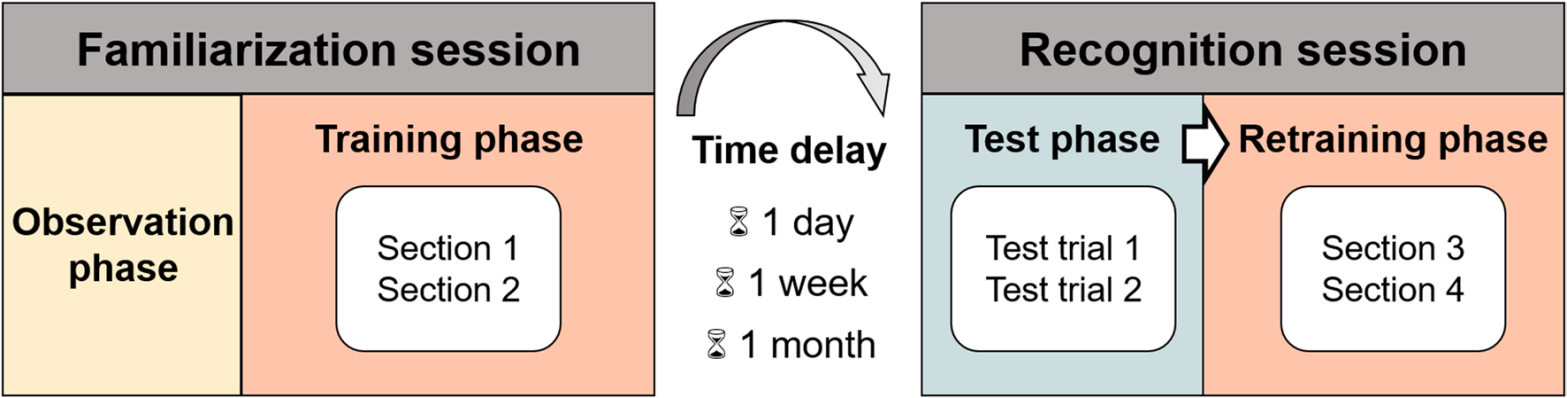
Scheme of the procedure; in Experiment 2 only one week delay was applied. The dog interacts with one UMO/human during the Training and Retraining phases (same UMO/human in both sessions). In the Test phase, four UMOs/two humans are presented in the room

All dogs were tested in two sessions: we introduced dogs to the UMO during the Familiarization session, and the Recognition session took place one day, one week or one month after the Familiarization session (Fig. 2). For a video about the procedure, see Online Resource 4.

##### Familiarization session

**Fig. 3:**
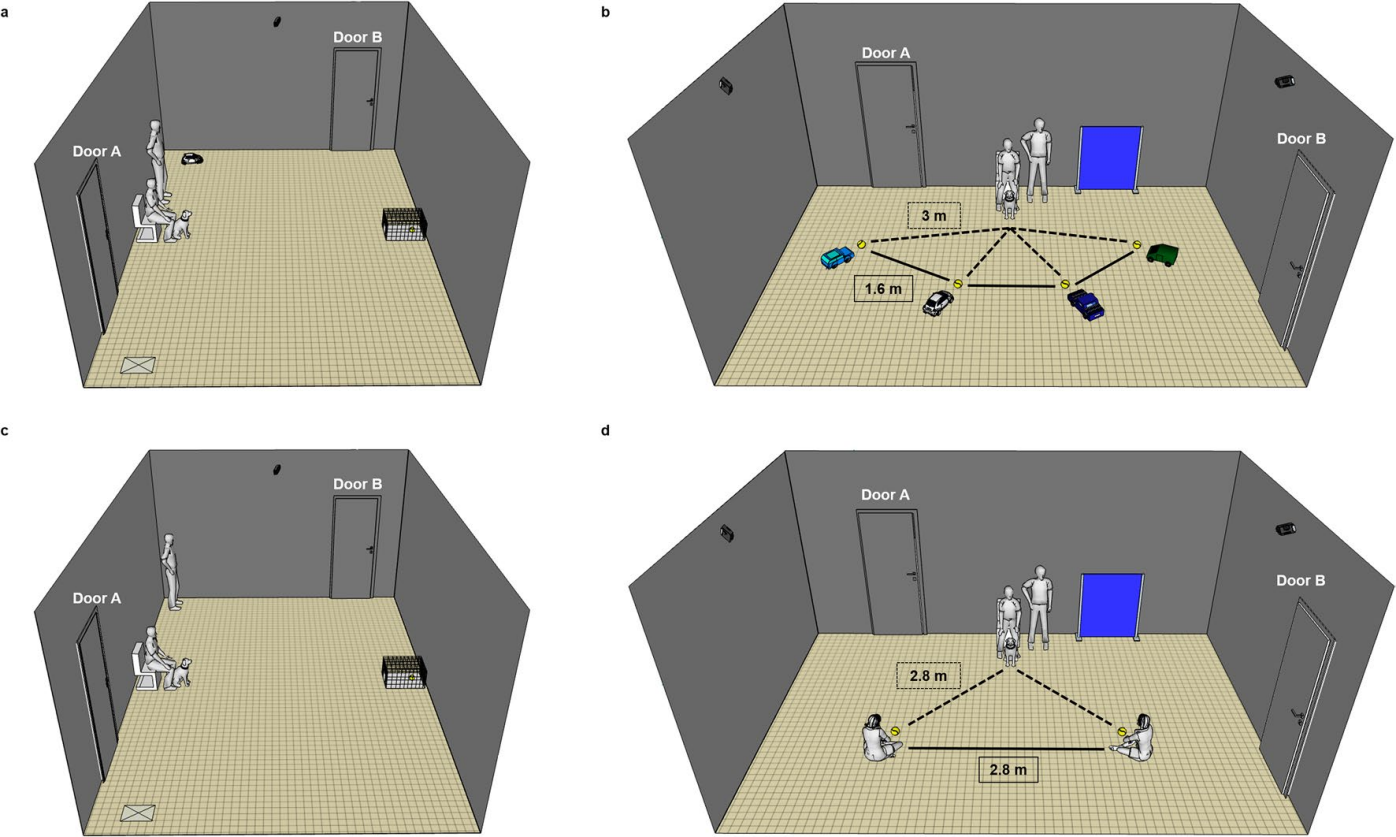
Experimental setup. Training and Retraining phases of **a** Experiment 1 and **c** Experiment 2; *X* marks the other starting position of the partner. Test phase of **b** Experiment 1 and **d** Experiment 2; note that in Test trial 1 there were no balls in front of the partners

*Observation phase*: Before the dog entered the room, the experimenters already placed a chair, the UMO, the cage and the plate in the room (Fig. 3a). The owner and the dog entered the room along with E1 and E2; the dog was allowed to explore the room while one of the experimenters gave the instructions to the owner. The owner sat on the chair and held the dog in front of him/her by the collar. E1 gave a tennis ball from outside of the room to E2 and then left the room. E2 played with the UMO shortly, to demonstrate to the dog that the UMO is able to move and push the ball, and to test in an indirect context whether the dog showed behavioural indications of stress in the presence of the UMO (e.g. hiding behind the owner, excessive barking at the UMO). During the playful interaction, E2 placed the ball in front of the UMO that pushed the ball to E2. This was repeated overall four times.

Following this, E1 entered the room and E2 gave him/her the ball. E2 stood on the left side of the chair. The owner released the dog and E1 played with the dog by throwing the ball 3–5 times to assess the way the ball could be retrieved from the dog. After this, E1 asked the owner to call the dog back and hold it in front of him/her.

*Training phase*: E1 called the dog’s attention by saying “Dog’s name! Look!” and bounced the ball on the ground. E1 placed the ball on the plate and put it inside the cage, attaching it to the magnet. E1 showed his/her empty hands to the dog and left the room. The owner released the dog on the signal of E2. The dog was allowed to move freely in the room and to try to obtain the ball. E2 avoided eye contact with the dog, and neither E2, nor the owner reacted to the dog’s behaviour. In the first trial of the Familiarization session, the UMO only started to move after 20 s. In all following trials, the UMO started to move either after 20 s or immediately when the dog looked at it. The UMO went inside the cage and retrieved the plate with the ball. The dog was allowed to retrieve the ball as soon as it could reach it.

After ball retrieval, a playing interaction started between the dog and the UMO facilitated by E2 if necessary. In case the dog placed the ball on the ground by itself, the UMO pushed the ball, and let the dog catch it. If the dog did not place the ball on the ground, E2 took the ball away from the dog and placed it in front of the UMO. Neither the owner, nor E2 threw the ball or engaged in other playful interactions with the dog. During the playing interaction, the UMO pushed the ball 2–7 times (in case of one dog the UMO pushed it more often, maximum of eleven times; mean pushes ± SD = 3.26 ± 1.24). The frequency of pushing the ball depended on the behaviour of the dog: if the dog placed the ball on the floor by itself, the UMO pushed it more often, but if the ball had to be taken away, the UMO pushed it less often to avoid inducing distress in the dog by repeatedly taking away the toy.

The playing interaction ended by the UMO moving back to its starting position, to the opposite side of the room from which it had started before the problem solving task (see Fig. 3a). E2 went back to her starting position. E1 entered the room and took the ball from the dog/E2. The above procedure was repeated at least five and a maximum of ten times (trials). After five trials, we stopped the experiment when the dog lost motivation or seemed to be stressed because the ball was continuously taken away (e.g. not grabbing the ball after the UMO pushed it or not willing to give the ball to E2 or its owner). We carried out 10 trials with eighteen dogs, 9 trials with two dogs, 8 trials with six dogs, 7 trials with seven dogs, 6 trials with eight and 5 trials with six dogs.

##### Recognition session

*Test phase*: Before the owner and the dog entered the room, the experimenters placed the chair, the occluder and a stopwatch in the room. The owner and the dog entered the room along with E1 and E2; the dog was allowed to explore the room while one of the experimenters explained the procedure to the owner. The owner sat on the chair and held the dog. E2 placed the occluder in front of the dog. E1 placed the four UMOs to their predetermined places (Fig. 3b). We counterbalanced the position of the familiar UMO within groups, and within subjects for which the familiar UMO had the same type of embodiment. The order of the other three, unfamiliar UMOs was also counterbalanced.

E1 left the room and E2 removed the occluder. Then the owner stood up and walked to the right corner with the dog on a leash. Starting from here, the owner led the dog in front of or behind the UMOs slowly. The owner did not stop at any of the UMOs, the dog had 2–3 s to assess each UMOs. After this, the owner sat back on the chair and held the dog in front of him/her. The owner released the dog to the indication of E2; the dog was allowed to move freely in the room (Test trial 1). After 30 s, E2 asked the owner to call the dog back, and placed the occluder in front of the dog again. E1 entered the room, placed one ball in front of each UMO and then left the room. E2 asked the owner to hold the dog in front of him/her, and she took the occluder away. The owner released the dog on the signal of E2; the dog was allowed to move freely in the room and could take away any of the balls (Test trial 2). Neither E2 nor the owner engaged in any interaction with the dog in either of the trials. After 30 s, E2 asked the owner to put the dog on the leash, and leave the room. After the owner left, the experimenters rearranged the room for the Retraining phase.

*Retraining phase*: We repeated the procedure of the Training phase to assess whether dogs’ behaviour changed after a delay, that is, irrespectively of remembering the familiar UMO, whether they remembered the behaviour of the UMO in general. This phase corresponded to the Training phase of the Familiarization session, except that (1) before the first trial, E1 played with the dog by throwing the ball 3–5 times to the dog (as in the Observation phase); (2) the UMO started to move within 20 s if the dog looked at it, even in the first trial; and (3) we carried out a maximum of eight trials.

##### Questionnaire

After the Recognition session, we sent a questionnaire to owners via email, which contained questions regarding the general playing habits of dogs (see Online Resource 1). For example, whether the dog prefers to play alone (chewing on the ball) or in interaction.

#### Behaviour and data analyses

Behaviour coding was carried out with Solomon Coder 19.08.02. (©András Péter: http://solomoncoder.com). Data were analysed using R software version 4.1.2 (R Core Team 2021) in RStudio version 1.4.1717 (RStudio Team 2021). We carried out backward model selection by using the drop1 function (except for mixed-effects Cox regression, for which this function is not available thus we used ANOVA for model comparison). Selection was based on the likelihood ratio test (LRT). Non-significant variables were excluded from the model, and we report the result of LRT before exclusion. In case of pairwise comparisons (“emmeans” package; Tukey correction), we report contrast estimates (β ± SE). Inter-coder reliabilities were carried out on a random subsample (20% of dogs). Inter-coder reliabilities were acceptable for all variables; for details see Online Resource 1.

##### Test phase

We coded the latency to first approach the UMO (s): from the moment the owner released the dog, until the dog approached the first UMO (within 0.5 m) (in case the dog did not approach any of the UMOs, we used the 30 s maximum time and indicated that the event did not happen). We used Cox regression (“survival” package) to analyse whether dogs approached the familiar UMO faster than the non-familiar UMOs (familiarity); and whether the time delay between the Familiarization and Recognition sessions (group), the type of UMO used as familiar (familiar UMO), placement of the UMOs (place), or the number of trials carried out during the Familiarization session (trial number; categorized as ten trials *vs* five-to-nine trials due to the differences in the subject number) had an effect on the latency of the dog’s first approach; and whether dogs showed preference for any of the UMOs (UMO-type). We also analysed whether dogs’ general preference to play alone vs in interaction with a human as reported by the owner, had an effect on the dog’s behaviour (playing style). We used separate models for Test trial 1 and 2. In both Test trials, there were dogs that did not approach any of the UMOs, thus familiarity, place and UMO-type could not be defined in these cases. In Test trial 1, eighteen dogs did not approach any of the UMOs (Day group, *N* = 6; Week group, *N* = 7; Month group, *N* = 5). Considering the large number of subjects that would be missing from the analysis, in Test trial 1 we did not include familiarity, place and UMO-type in the model. In Test trial 2, only four dogs did not approach any of the UMOs. Considering that testing whether there is an interaction between group and familiarity was an important aspect, the latter of which cannot be defined in these cases, we left the data of these dogs out from the model (Day group, *N* = 2; Week group, *N* = 1; Month group, *N* = 1).

We also analysed whether dogs chose first the familiar UMO above chance level (binomial test; chance level 0.25). For this analysis, we included only subjects that approached at least one of the UMOs.

We also investigated the within-trial dynamics of looking at the UMOs in the two Test trials separately by constructing looking-time curves (Python 3.7.6 in Jupyter Notebook 6.0.3). We determined for every 0.2 s the proportion of dogs looking at any of the UMOs. To capture overall trends, we applied linear regression to the data and provide the slope of the regression line (β ± SE). Considering that in Test trial 2 it could not be determined whether the dog looks at the partner or the ball, here looking at the partner included looking at the ball as well.

##### Comparison of the training and retraining phases

We coded the latency of dogs’ first look at the UMO (s): from the moment the owner released the dog, until the UMO started to move (in case the dog did not look at the UMO, we used the 20 s maximum time and indicated that the event did not happen). We tested whether the time delay between the Familiarization and Recognition sessions (group); the type of UMO used as familiar (familiar UMO); and the number of trials carried out during the Familiarization session (Trial number; see above) had an effect on the latency of first look. We also compared whether the latency of first look changed between the Training and Retraining phases (Training). We also analysed the change in this behaviour on a subtler scale, and thus for another analysis we separated the first and second part of the Training and Retraining phases within each dog (Section; Training phase: Sections 1 and 2; Retraining phase: Sections 3 and 4; e.g. in case a dog had 8 trials in the Training phase, Section 1 was trials 1–4, and Section 2 was trials 5–8). This way we could analyse whether dogs’ behaviour was different, for example, when they interacted with the UMO first (Section 1) vs meeting the UMO again after the specific period of time (Section 3). We used mixed-effects Cox regression (“coxme” package) to analyse the data (all dogs were assigned with an ID number that was used as random variable).

We also analysed whether dogs put the ball down to the UMO (binary variable) or to any of the humans (binary variable) (in every trial ‘1’ indicates if the dog put the ball down, and ‘0’ if not). Behaviour was defined as the dog putting the ball down without a verbal command or hand signal while orienting toward the UMO/human. For the analysis of this behaviour and results, see Online Resource 1.

### Results

#### Test phase

First approach of dogs was on chance level both in Test trial 1 (*p* = 0.398) and Test trial 2 (*p* = 0.725) (binomial test; chance level 0.25). Regarding dogs’ behaviour in the different groups, their choice was on chance level in all groups both in Test trial 1 (day group: *p* = 0.466; week group: *p* = 0.700; month group: *p* = 0.312) and in Test trial 2 (day group: *p* = 0.748; week group: *p* = 0.550; month group: *p* = 1.000).

The time elapsed between the two sessions did not have an effect on the latency of dogs’ first approach of the UMOs in Test Trial 1, i.e. when there were no balls in front of the UMOs (Group: ${\chi }_{2}^{2}$ = 1.831, *p* = 0.400). The playing style by group interaction had no effect on dog’s behaviour either (Cox regression, LRT: Group x Playing style, ${\chi }_{2}^{2}$ = 1.286, *p* = 0.526). Although dogs’ playing style had a main effect on the latency of approach (Playing style: ${\chi }_{1}^{2}$ = 5.110, *p* = 0.024), pairwise comparison failed to find a significant difference between dogs that prefer to play in interaction vs dogs that play alone (play in interaction vs play alone: β ± SE = 0.408, *p* = 0.275). The type of the familiar UMO and the number of trials carried out during the Familiarization session did not have an effect on the latency of approaching the first UMO (Familiar UMO: ${\chi }_{3}^{2}$ = 1.582, *p* = 0.663; Trial number: ${\chi }_{1}^{2}$ = 0.007, *p* = 0.935).

**Fig. 4:**
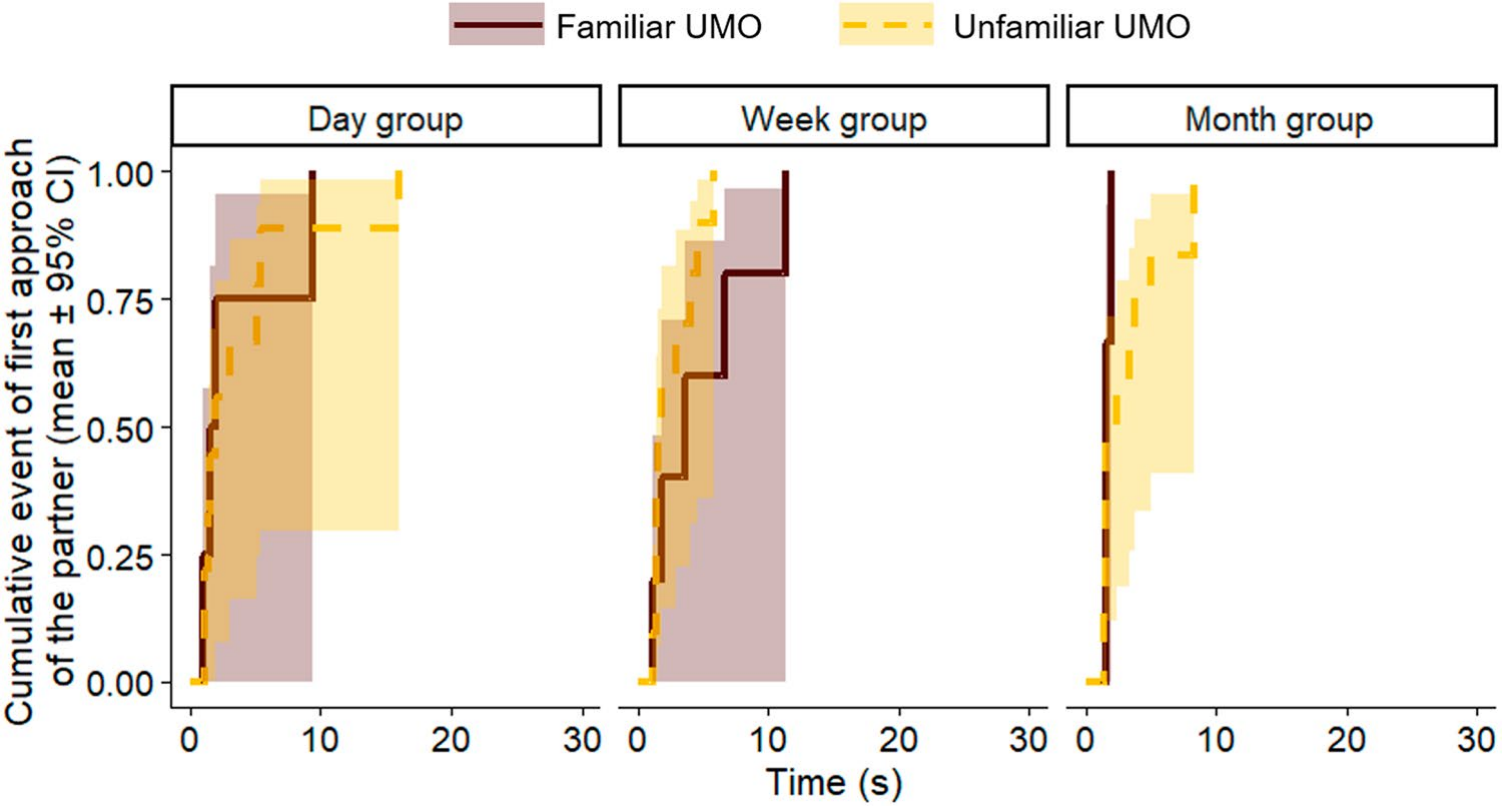
Latency to approach the first UMO in Test trial 2 (four-way choice with balls in front of the UMOs). Approaching the familiar or unfamiliar UMO depending on the time passed between the Familiarization and the Recognition sessions (Cox regression)

In Test Trial 2, when there was a ball in front of each UMO, neither the three-way interaction of familiarity, group and playing style (Cox regression, LRT: Familiarity × Group × Playing style, ${\chi }_{2}^{2}$ = 0.198, *p* = 0.906), nor any of the two-way interactions showed significant effect on the latency of dogs’ approach of the first UMO (Familiarity × Group: ${\chi }_{2}^{2}$ = 2.258, *p* = 0.323; Familiarity × Playing style: ${\chi }_{1}^{2}$ = 0.005, *p* = 0.945; Group × Playing style: ${\chi }_{2}^{2}$ = 2.000, *p* = 0.368) (Fig. 4). Dogs’ did not approach the familiar UMO sooner than the unfamiliar UMOs (Familiarity: ${\chi }_{1}^{2}$ = 0.150, *p* = 0.698), and the time elapsed between the two sessions did not have an effect on dogs’ behaviour either (Group: ${\chi }_{2}^{2}$ = 0.403, *p* = 0.818). Dogs preferring to play in interaction did not approach the UMOs sooner than dogs that prefer solitary play (Playing style: ${\chi }_{1}^{2}$ = 2.685, *p* = 0.101). Further, the type of the familiar UMO and the number of trials carried out during the Familiarization session did not have an effect on the latency of approaching the first UMO (Familiar UMO: ${\chi }_{3}^{2}$ = 6.322, *p* = 0.097; Trial number: ${\chi }_{1}^{2}$ = 0.887, *p* = 0.346). Dogs did not show preference for any of the UMOs or the places the UMOs were placed in Test trial 2 (UMO-type: ${\chi }_{3}^{2}$ = 3.567, *p* = 0.312; Place: ${\chi }_{3}^{2}$ = 6.475, *p* = 0.091).

**Fig. 5:**
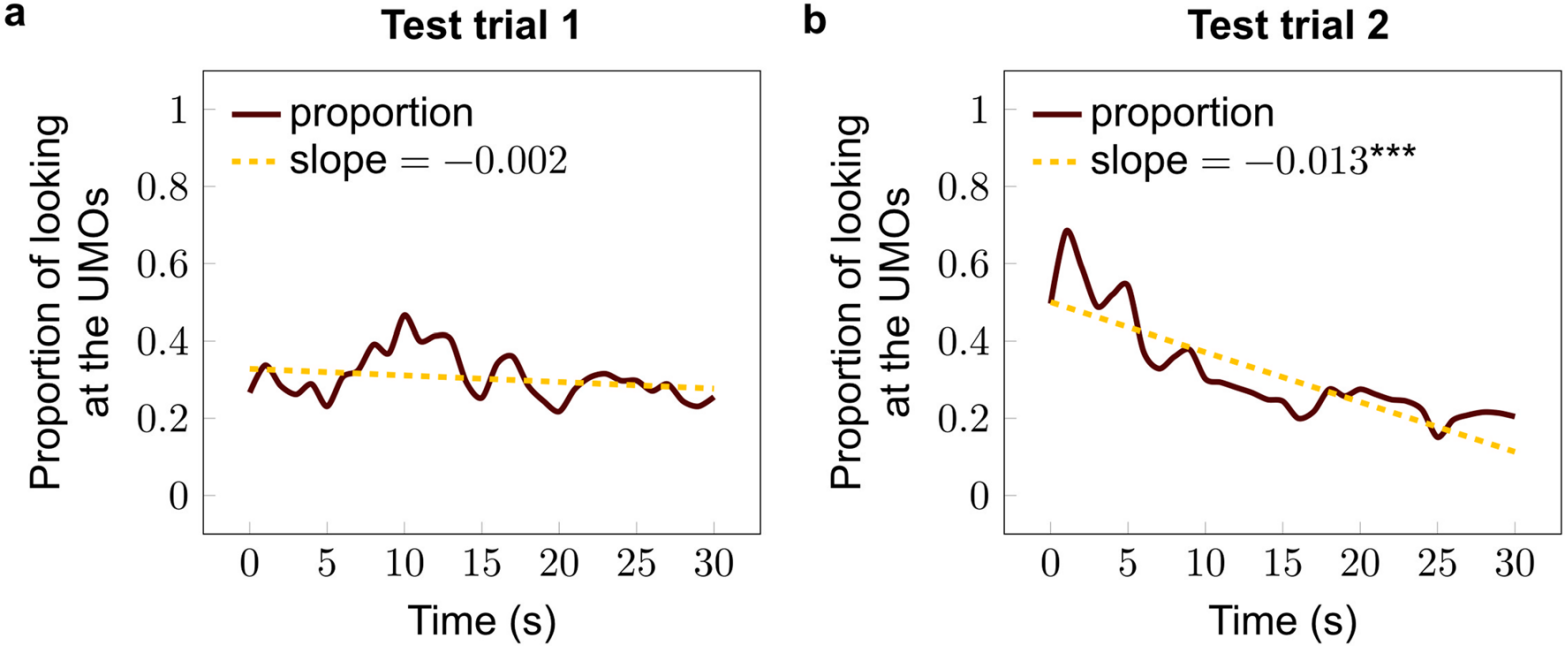
Dynamics of the duration of looking at the UMOs during **a** Test trial 1 (without ball); **b** Test trial 2 (with balls) (Linear regression)

Dynamics of dogs’ looking time toward the UMOs did not change during Test trial 1 (Linear regression, β ± SE = − 0.002 ± 0.001; *p* = 0.152) (Fig. 5a); however, their look toward the UMOs decreased significantly during Test trial 2 (β ± SE = − 0.013 ± 0.002; *p* < 0.001) (Fig. 5b).

#### Comparison of the training and retraining phases

**Fig. 6:**
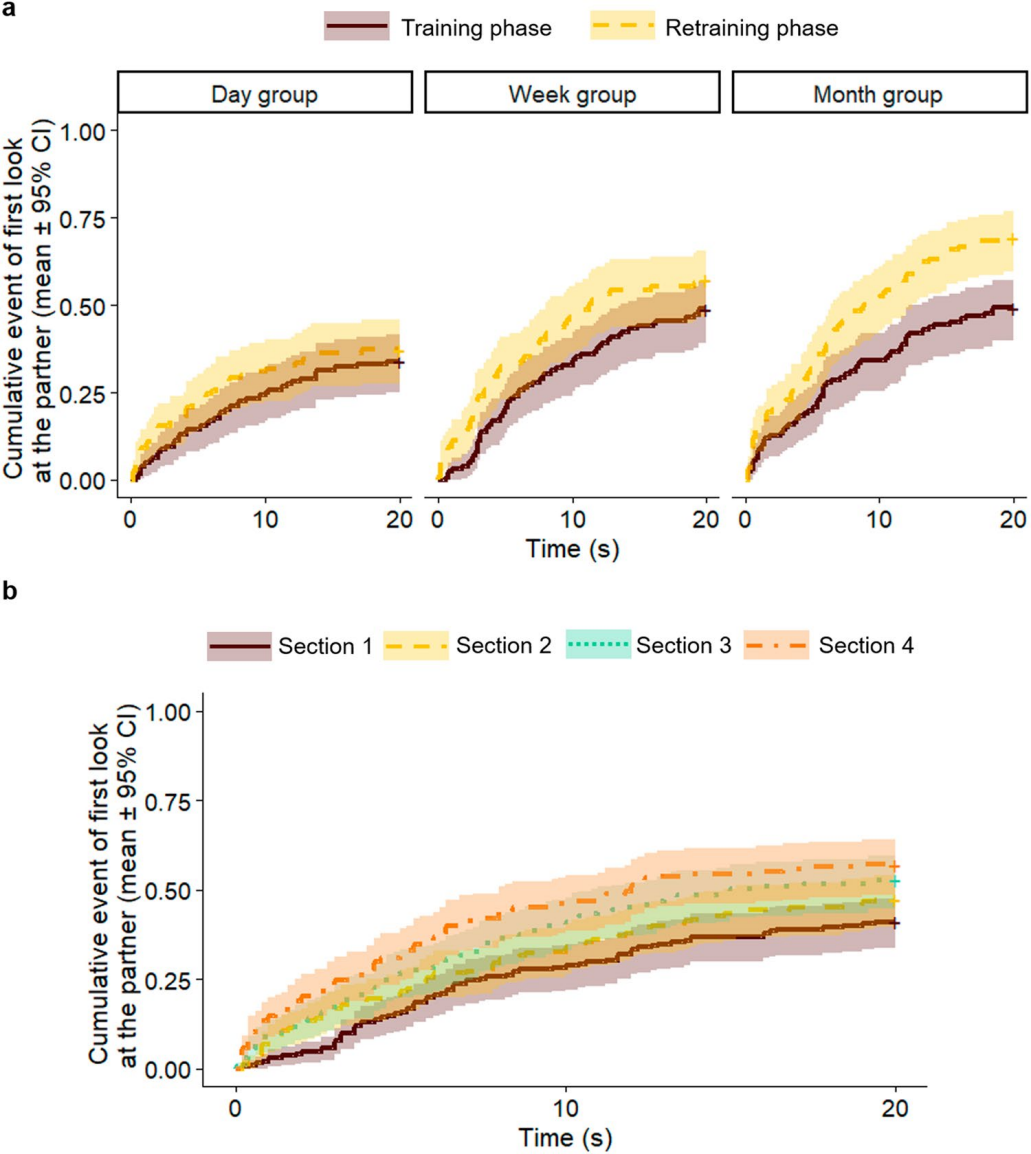
Latency to look at the UMO in the Training and Retraining phases of the different groups; **a** in the two sessions; **b** in the four sections (mixed-effects Cox regression)

The interaction of group and session had no overall effect on the latency of first look at the UMO during the problem solving task (Mixed-effects Cox regression, LRT: Group × Training, ${\chi }_{2}^{2}$ = 1.469, *p* = 0.480) (Fig. 6a). Overall, it did not have an effect whether dogs were retested after one day, one week or a month (Group, ${\chi }_{2}^{2}$ = 4.930, *p* = 0.085). However, dogs looked at the UMO sooner in the Training, than in the Retraining phase (Training, ${\chi }_{1}^{2}$ = 14.096, *p* < 0.001; Training vs Retraining phases, β ± SE = − 0.409 ± 0.109; *p* < 0.001). In a further analysis, we found that latency of looking at the UMO varied between sections as well (Mixed-effects Cox regression, LRT: Section, ${\chi }_{3}^{2}$ = 19.383, *p* < 0.001) (Fig. 6b). Pairwise comparison revealed that dogs’ behaviour did not change significantly within the Training phase of the Familiarization session or within the Retraining phase of the Recognition session (Section 1 vs 2:, β ± SE = -0.278 ± 0.157, *p* = 0.286; Section 3 vs 4: β ± SE = − 0.224 ± 0.150, *p* = 0.442). However, dogs looked at the UMO sooner at the beginning of the Retraining phase compared to the beginning of the Training phase (Section 1 vs 3: β ± SE = − 0.437 ± 0.155, *p* = 0.025), and there was no difference in the latency of look between the end of the Training vs the beginning of the Retraining phases (Section 2 vs 3: β ± SE = − 0.158 ± 0.153, *p* = 0.729).

Neither the type of the familiar UMO, nor the number of trials carried out during the Familiarization session had an effect on dogs’ behaviour (familiar UMO, ${\chi }_{3}^{2}$ = 1.636, *p* = 0.651; trial number, ${\chi }_{1}^{2}$ = 0.0007, *p* = 0.979).

### Discussion

Dogs did not prefer the specific UMO that had previously helped them to obtain an unreachable ball and had shown playful behaviour. Thus, we could not find evidence that dogs show IR of an UMO even after a day, using the present experimental protocol. In contrast, dogs displayed similar behaviour toward the UMO at the beginning of the Recognition session as at the end of their first encounter. Thus, dogs did not seem to have recognized the specific individual they had experience with, but they remembered the behaviour of the UMO even a month following a single, short interaction.

In the present experiment, dogs were exposed to the UMO in two types of social interactions, but they only met the UMO for a short time (about 30 min) at one occasion. It is likely that including other social contexts, increasing the time spent with the UMO or the frequency of encounters within a period, facilitates the recognition of the specific UMO by learning about its individual cues.

Dogs initiated playful interaction with the UMO, but they were more likely to invite a human to play. Dogs that in general prefer solitary play were less likely to put the ball down for either of the partners, thus preference for solitary play vs playful interaction seems to be an individual trait of the dog independent of the partner (see Online Resource 1).

One may suggest that lack of recognition of the individual UMO is specific to the artificial agent due to the novelty of the partner. However, we have no information what type of and how much experience is needed with an unfamiliar human to identify them individually, especially considering long-term memory of the individual. Thus, it is unclear whether lack of recognition of the familiar partner (1) was related specifically to the UMO, or (2) our method was not sensitive enough for detecting it.

First, the UMO is a novel, artificial partner thus dogs first had to recognize them as an animate and interactive agent and learn about its behaviour (e.g. it can help to reach a reward). Also, the UMO has several limitations regarding its capabilities, for example, it could not talk to the dog, ask/take away the ball and throw the ball in the same way as a typical interactive human partner which could influence the quality of the interaction. Due to the limitations of the UMO, an extra human (E2) had to be involved to facilitate the dog-UMO interaction that could distract the dogs’ attention from the UMO during playing. Although one may suggest that the UMO is merely an object, based on previous findings, dogs engage in interaction with these artificial agents in various situations (including the one applied in the present study) and display similar social behaviour toward the UMO as toward a regular social partner, a human (see “Introduction”).

Second, some aspects of our procedure might not allow the emergence of IR, irrespectively, whether it is a usual (e.g. human) or novel (UMO) partner. For example, the short duration of the interaction and introducing the partner in only two types of contexts might be insufficient to notice and remember unique characteristics of the partner after a longer delay. Also, having to choose between four different partners might present a too complex situation and/or dogs are not motivated to display specific behaviour toward the familiar partner despite its recognition.

## Experiment 2

Following the findings of Experiment 1, here we wanted to explore dogs’ behaviour when they face the same situation but with human partners instead of UMOs. We applied the same problem-solving and playing situation, but changed some details of the general procedure based on the findings of Experiment 1 to improve dogs’ chance of recognizing the familiar partner. (a) The results of Experiment 1 showed that playing style of dogs influenced their behaviour at least during the playing interaction, thus we only invited dogs that prefer to play in interaction with humans based on the owner’s opinion. (b) Considering that dogs displayed similar behaviour independently of the time elapsed between the two occasions, here we only tested dogs with a week delay. We chose this option to be able to test dogs’ behaviour over a longer period, but improve the chance of remembering the partner. (c) To make the choice easier and thus be able to find out whether the choice test is appropriate to study IR in this scenario, here we applied a two-way, instead of a four-way choice test. (d) Also, we did not need an experimenter facilitating the playing interaction of the dog and the partner thus we could exclude this potentially confounding factor. (e) In the Retraining phase, we had ten trials similarly to the Training phase (cf. Experiment 1 where only eight trials were carried out in the Retraining phase).

Although studies investigating IR of humans mainly focused on the recognition of the owner (e.g. Huber et al. 2013), it is assumed that dogs are able to individually identify other humans as well. Thus, we hypothesised that dogs would remember the familiar human that helped them in a problem solving task and played with them. Irrespectively of the recognition of the specific individual, we expected that dogs would remember the interactive behaviour of the human partner after a week, considering that dogs encounter similar situations (humans helping to solve a problem) in their daily lives.

### Methods

#### Subjects

We included dogs above one year of age that could be motivated with a ball, based on the owner’s opinion. We invited dogs that preferred to play in interaction rather than solitary play. We tested 22 dogs, but had to exclude two dogs: one dog showed distress in the room and one dog was not motivated by the ball. Twenty dogs remained in the final analyses after exclusion (different breeds, 13 females; mean ± SD age: 5.0 ± 2.4 years) (for more details see Online Resource 3). There was no overlap between the subjects in Experiment 1 and 2.

#### Test partner and apparatus

The test partners were two adult females (BL and ZG), we counterbalanced between dogs whether BL or ZG was the familiar partner. Irrespectively of whether they played the familiar (FH) or unfamiliar human (UH) partner, BL wore black shoes, black pants, black long-sleeved shirt and a black disposable surgical mask, and had long dark brown hair, whereas ZG wore black and white shoes, burgundy pants, white long-sleeved shirt and light blue mask, and had a long reddish blonde hair. The hair of the familiar partner was always in a ponytail, whereas the hair of the unfamiliar partner was always down. Only one E was present throughout the experiment (same as E2 in Experiment 1; JA).

Dogs were tested in the same room as subjects in Experiment 1 and all experimental equipment was the same. The only difference was that the opening of the cage was smaller to prevent small-sized dogs to go inside (W × H: 20 cm × 16 cm), instead of the wooden-cartonplast barrier used in Experiment 1.

#### Procedure

All dogs were tested in two sessions: we introduced dogs to the familiar human during the Familiarization session, and the Recognition session took place one week after the Familiarization session (Fig. 2). The procedure was the same as in Experiment 1, thus here we only describe the differences in the specific phases. For a video about the procedure, see Online Resource 4.

##### Familiarization session

*Observation phase*: Same as in Experiment 1.

*Training phase*: The procedure was essentially identical to that described in Experiment 1, with few small changes (see the experimental setup on Fig. 3c). (1) After E hid the ball inside the cage, she left the room and owners were instructed to count until 10 and then release the dog (no experimenter was present in the room during the problem-solving and playing part). (2) FH looked down during the trial and looked at the dog on her periphery to avoid eye contact. (3) After obtaining the ball from the cage, FH threw the ball immediately to the dog. (4) Regarding the playing interaction, FH asked/took the ball away from the dog. During the playing interaction, FH could talk to the dog and engage in a conversation with the owner to create a more natural situation. During the playing interaction, the human partner threw the ball 2–7 times (in case of one dog in one trial, and one dog in two trials the partner threw the ball 8 times, and in case of one dog the partner threw the ball 9 times in three trials) (mean throws ± SD = 4.97 ± 1.09). The frequency of throwing the ball depended on the behaviour of the dog: if the dog placed the ball on the floor by itself, the partner threw it more often, but if the ball had to be taken away, the partner threw it less often to avoid inducing distress in the dog by repeatedly taking away the toy. However, here we invited dogs that prefer to play in interaction and, thus, were more likely to put the ball down by themselves.

The procedure was repeated at least five and a maximum of ten times (trials). After five trials, we stopped the experiment when the dog lost motivation. We carried out 10 trials with fifteen dogs, 9 trials with one dog, 8 trials with two dogs, 7 trials with one dog, and 5 trials with one dog.

##### Recognition session

*Test phase*: Only two human partners (FH and UH) were introduced simultaneously to dogs. After the owner sat on the chair and held the dog in front of him/her, E placed the occluder in front of the dog and FH and UH entered the room (Fig. 3d). FH and UH sat down on the ground with crossed legs, equal distances from each other and the dog’s starting position. FH and UH held their hands in front of them with their palms upward so the dog can assess that they do not have anything their hands. We counterbalanced the position of FH and UH between dogs.

E removed the occluder and stood next to the chair on its left side. Then the owner stood up and with the dog on leash, walked to one of the human partners and then to the other one before sitting back. The dog had 2–3 s to assess each human partners. We counterbalanced between dogs whether the human introduced first was FH or UH, and whether the firstly introduced partner was sitting on the left or right side (from the dogs’ point of view). After exploring both partners, the owner sat back to the chair and held the dog in front of him/her. From here, we applied the same procedure as described in Experiment 1.

*Retraining phase*: This phase corresponded to the Training phase of the Familiarization session, except that (1) before the first trial, E played with the dog by throwing the ball 3–5 times to the dog (as in the Observation phase); and (2) FH started to move within 20 s if the dog looked at it, even in the first trial.

#### Behaviour and data analyses

Behaviour coding was carried out with Solomon Coder 19.08.02. (©András Péter: http://solomoncoder.com). Data were analysed using R software version 4.1.2 (R Core Team 2021) in RStudio version 1.4.1717 (RStudio Team 2021). The statistical analyses were carried out in the same way as in Experiment 1. Inter-coder reliabilities were acceptable for all variables, except for putting the ball down for the partner or owner; for details see Online Resource 1.

##### Test phase

We coded the latency to the first approach of the partner (s): from the moment the owner released the dog, until the dog approached the first human partner (within 0.5 m) (in case the dog did not approach any of the partners, we used the 30 s maximum time and indicated that the event did not happen). We used Cox regression (“survival” package) to analyse whether dogs approached the familiar human faster than the unfamiliar human (familiarity) or displayed preference to the left or right side (place). We also analysed whether the place where the familiar partner was sitting (familiar place), the human partner or side approached first during the introduction (intro human and intro side, respectively) or whether BL or ZG was the familiar human (familiar human) had an effect on the latency of the dog’s first approach. Considering that only in the case of five dogs less than 10 trials was carried out in the Training phase of the Familiarization session, we did not analyse the effect of trial number here. We used separate models for Test trial 1 and 2. In both Test trials, there were dogs that did not approach any of the partners, thus familiarity and place could not be defined in these cases. Seven dogs in Test trial 1, and four dogs in Test trial 2 did not approach any of the partners. The effect of familiarity and place was only tested in case of dogs that approached at least one of the partners; all other variables were tested including all subjects.

We also analysed whether dogs chose first the familiar partner above chance level (chance level 0.5). For this analysis, we included only subjects that approached at least one of the partners.

We also investigated the within-trial dynamics of looking at the partners in the two Test trials. In Test trial 2, looking at the partners included looking at the balls placed in front of the partners as well, because in case of the UMOs, it was not possible to discriminate between dogs looking at the UMO or the ball. Analysis was carried out the same way as described in Experiment 1.

##### Comparison of the training and retraining phases

We coded the latency of dogs’ first look at the human partner (s): from the moment the owner released the dog, until the partner started to move (in case the dog did not look at the the partner, we used the 20 s maximum time and indicated that the event did not happen). We compared whether the latency of first look changed between the Training and Retraining phases (Training). We also analysed the change in this behaviour on a subtler scale, and thus for another analysis we separated the first and second part of the Training and Retraining phases within each dog (section; see “Experiment 1”). We also tested whether the identity of the partner (BL vs ZG) had an effect on dogs’ latency of first look at the partner. We used mixed-effects Cox regression (“coxme” package) to analyse the data (all dogs were assigned with an ID number that was used as random variable).

### Results

#### Test phase

**Fig. 7:**
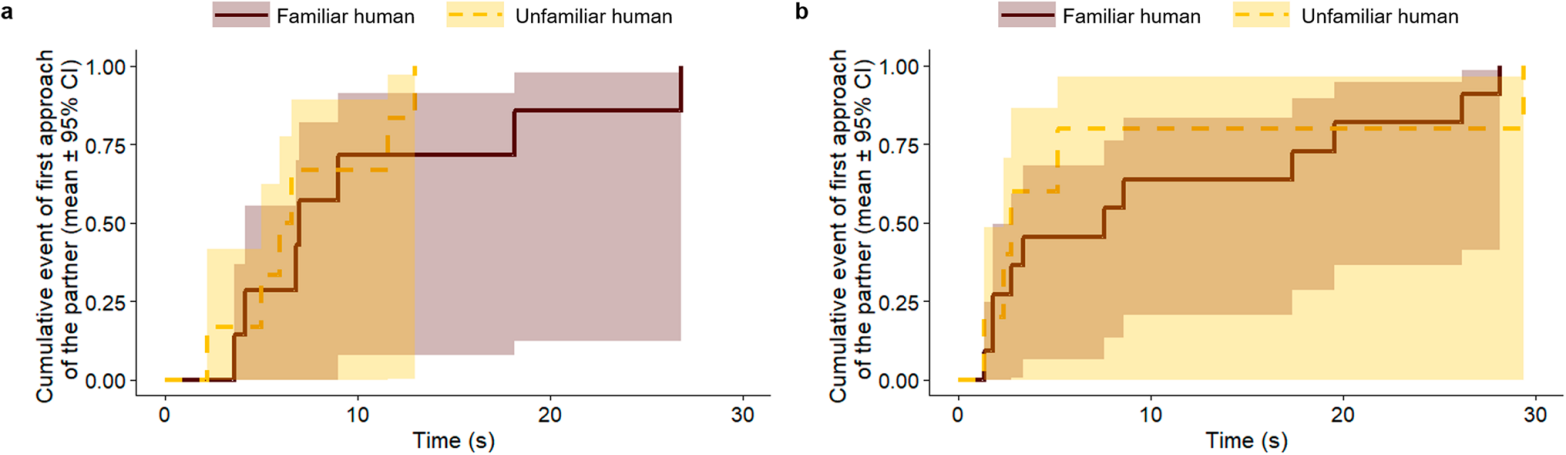
Latency to approach the first human partner in **a** Test trial 1 (without ball) and **b** Test trial 2 (with ball), in Experiment 2 (Cox regression)

First approach of dogs was on chance level both in Test trial 1 (*p* = 1.000) and Test trial 2 (*p* = 0.210) (binomial test; chance level 0.5). Neither in Test Trial 1, nor in Test trial 2 dogs approached the familiar human partner sooner than the unfamiliar partner (Familiarity: Test trial 1, ${\chi }_{1}^{2}$ = 0.840, *p* = 0.359; Test trial 2, ${\chi }_{1}^{2}$ = 0.212, *p* = 0.645) (Fig. 7). Dogs were also not likely to approach the partner either on the right or on the left side (Place: Test trial 1, ${\chi }_{1}^{2}$ = 0.229, *p* = 0.632; Test trial 1, ${\chi }_{1}^{2}$ = 0.229, *p* = 0.632). None of the other variables influenced dogs’ latency of first approach (Intro side: Test trial 1, ${\chi }_{1}^{2}$ = 0.022, *p* = 0.881; Test trial 2, ${\chi }_{1}^{2}$ = 1.045, *p* = 0.307; Intro human: Test trial 1, ${\chi }_{1}^{2}$ = 0.196, *p* = 0.658; Test trial 2, ${\chi }_{1}^{2}$ = 0.416, *p* = 0.519; Familiar human: Test trial 1, ${\chi }_{1}^{2}$ = 3.747, *p* = 0.052; Test trial 2, ${\chi }_{1}^{2}$ = 3.573, *p* = 0.059; Familiar place: Test trial 1, ${\chi }_{1}^{2}$ = 0.003, *p* = 0.957; Test trial 2, ${\chi }_{1}^{2}$ = 0.078, *p* = 0.780).

**Fig. 8:**
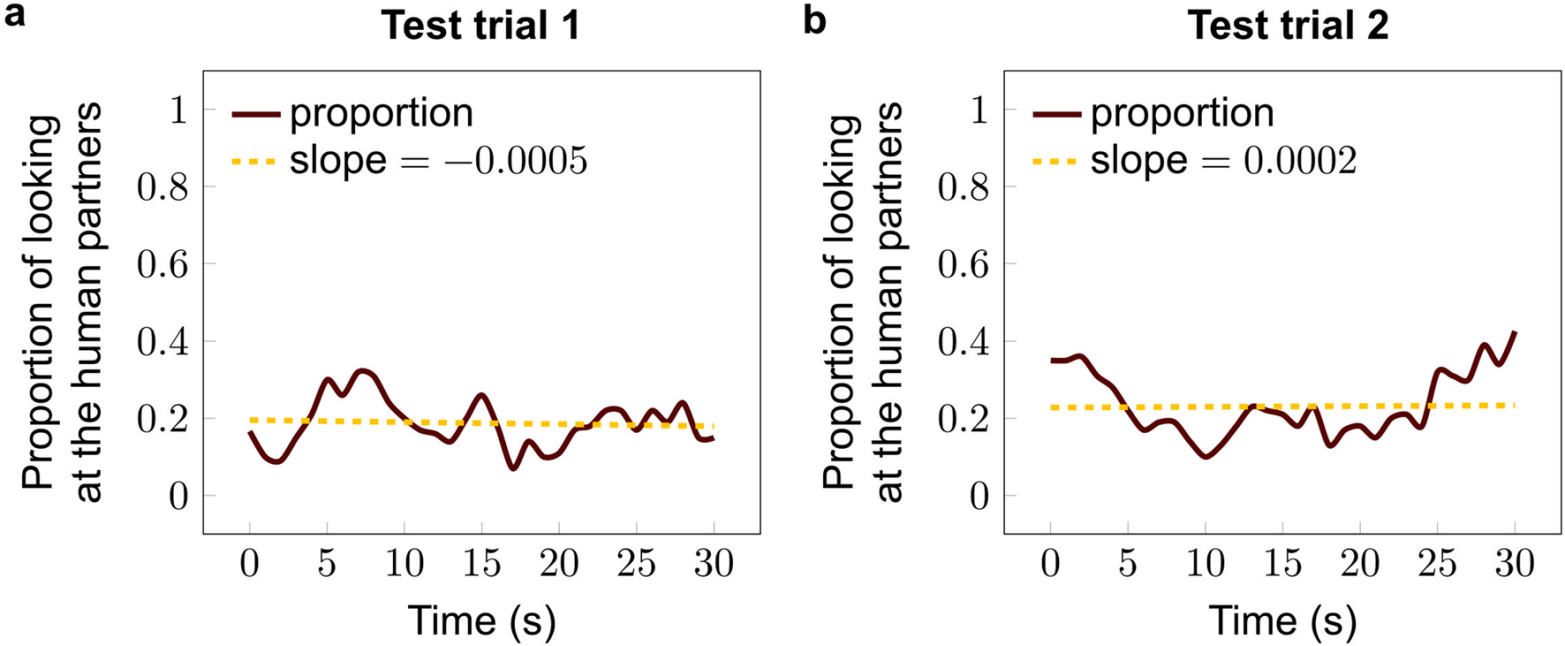
Dynamics of the duration of looking at the human partners during **a** Test trial 1 (without ball); **b** Test trial 2 (with balls) in Experiment 2 (Linear regression)

Dynamics of dogs’ looking time toward the partners did not change during Test trial 1 (Linear regression, β ± SE = − 0.0005 ± 0.001; *p* = 0.705) or Test trial 2 (β ± SE = 0.0002 ± 0.002; *p* = 0.914) (Fig. 8).

#### Comparison of the training and retraining phases

**Fig. 9:**
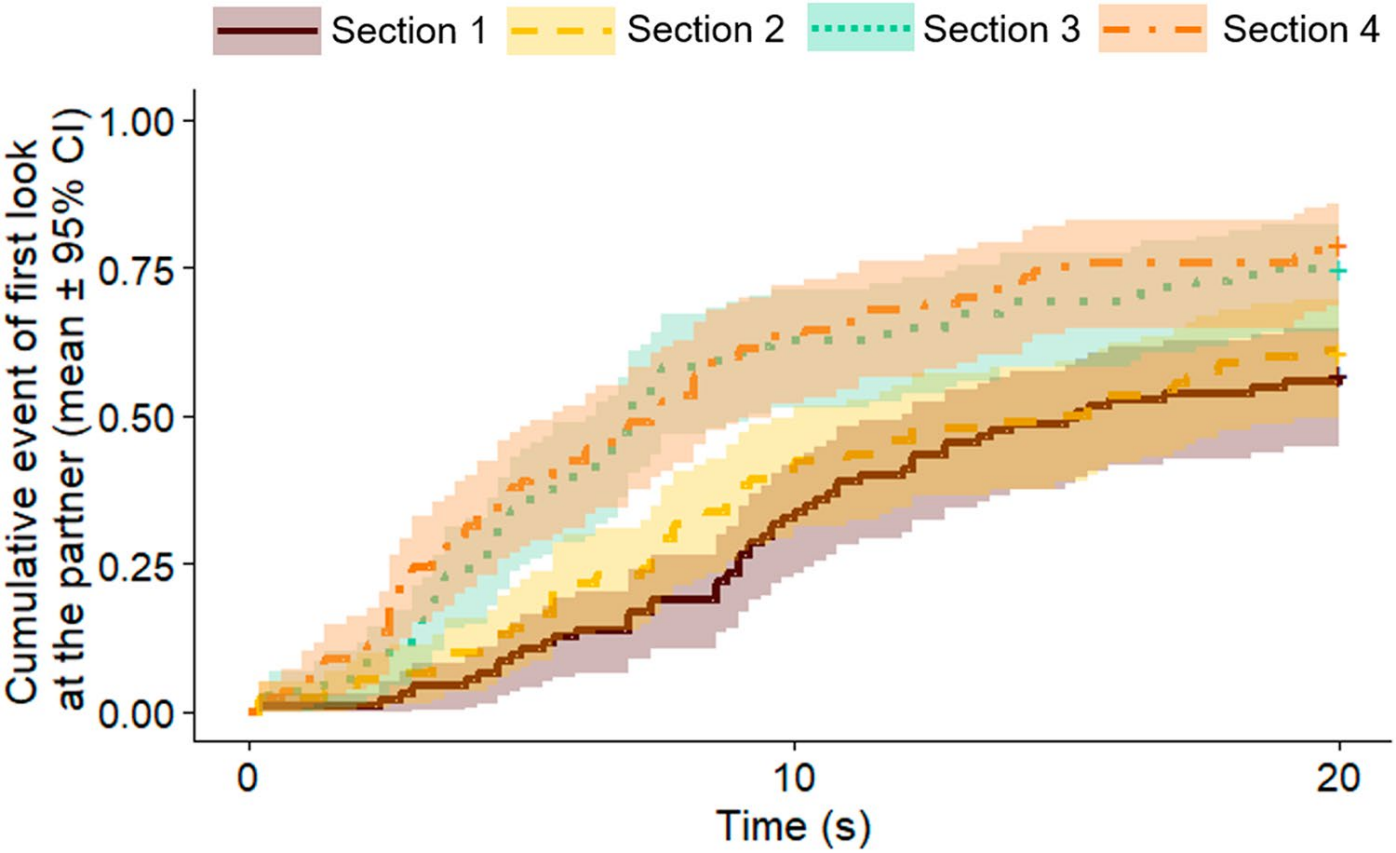
Latency to look at the human partner in the four sections of the Training and Retraining phases, in Experiment 2, that is, in the first and second half of the Training phase (Section 1 and 2), and in the first and second half of the Retraining phase (Section 3 and 4) (mixed-effects Cox regression)

Dogs looked at the human partner sooner in the Retraining phase of the Recognition session, than in the Training phase of the Familiarization session (Training, ${\chi }_{1}^{2}$ = 38.216, *p* < 0.001; Familiarization vs Recognition, β ± SE = − 0.837 ± 0.136; *p* < 0.001). In a further analysis, we found that latency of looking at the partner varied between sections as well (Mixed-effects Cox regression, LRT: Section, ${\chi }_{3}^{2}$ = 39.348, *p* < 0.001) (Fig. 9). Pairwise comparison revealed that dogs’ behaviour did not change significantly within the Training phase or within the Retraining phase (Section 1 vs 2: β ± SE = − 0.189 ± 0.192, *p* = 0.760; Section 3 vs 4: β ± SE = − 0.069 ± 0.172, *p* = 0.980). However, dogs looked at the partner sooner at the beginning of the Retraining phase compared to the beginning of the Training phase (Section 1 vs 3: β ± SE = − 0.894 ± 0.190, *p* < 0.001). Dogs also looked sooner at the partner at the beginning of the Retraining phase than at the end of the Training phase (Section 2 vs 3: β ± SE = − 0.706 ± 0.187, *p* < 0.001). The identity of the human partner (LB vs ZG) did not influence dogs’ behaviour (familiar human, ${\chi }_{1}^{2}$ = 0.134, *p* = 0.714).

### Discussion

Our findings show that dogs do not show specific behaviour toward a familiar human after a week delay following a short social interaction. However, they seem to remember the behaviour of the human in general. Although previous findings suggest that dogs are able to recognize their owner (Adachi et al. 2007; Huber et al. 2013), this manifests after excessive experience and owners thus represent a unique category within humans. Thus, in contrast to the common belief, dogs need more time and experience with a human individual to develop a long-term memory about him/her specifically. These results suggest that failure in recognizing the familiar UMO was not specific to the agent, rather dogs did not gain enough experience during the short interaction in Experiment 1.

## General discussion

Overall, our results indicate that dogs do not display preference to a specific individual irrespectively of whether it is a usual (human) or an unusual (UMO) agent. We propose that dogs need more experience with a specific partner to be able to develop a long-term memory about them. Here the UMO was a novel interactive partner that visually did not resemble any social partner the dog had interacted with during development. We only familiarized dogs with a single specific UMO, and due to the lack of comparison with another one during the first occasion, dogs might have learnt some features of the specific individual that are shared among UMOs (e.g. size or angular shape) instead of its individually unique visual characteristics (cf. perceptual learning; Lu et al. 2011). Thus, our dogs might have generated CLR of the UMOs. One solution to disentangle the question of CLR vs IR of UMOs would be to familiarize dogs with all partners (having different experiences with them), thus they would have similar familiarity with the dog. However, dogs did not discriminate between the human partners either, and considering that family dogs have extensive experience with humans, this explanation is less likely to be responsible for dogs’ behaviour in Experiment 2.

It is likely that IR is often based on multiple sensory channels, including olfactory, auditory or visual input (see “Introduction”). In the present study, dogs could rely mainly on visual differences between the familiar and unfamiliar partners (note that although human partners were talking during the play, they were silent in the Test phase); however, multiple cues might facilitate the recognition of the specific partner even in case of limited experience. One may suggest that our results reflect that dogs were unable to distinguish between the UMOs (and humans) visually. However, dogs are able to discriminate between black and white stimuli, different shades of grey, and between objects based on size (e.g. Araujo et al. 2004; Burman et al. 2011; Milgram 2003; Tapp et al. 2003). Dogs also react to changes in colour and size of an object (Müller et al. 2011; Pattison et al. 2013) and to changing an object to another (kind-relevant change; Johnston et al. 2021). Word learning studies carried out with dogs further suggest that they are able to discriminate between hundreds of objects (e.g. Dror et al. 2021; Kaminski et al. 2008). Thus, we expected that dogs are able to discriminate between UMOs based on their visual characteristics.

We also cannot exclude that our method might not be appropriate to test whether dogs individually recognize a partner. Choice tests are frequently used to study CLR or IR (e.g. Brajon et al. 2015; Engelmann et al. 1995; Madeira and Oliveira 2017), but the measured indicative behaviour varies across species and contexts. Thus, depending on the context researchers can measure, for example, aggression, preference, avoidance, etc. Here we expected that dogs either (1) approach first the familiar partner because of their previous positive interaction, or because they expect it/her to retrieve a ball (when no ball was present) and engage in a playing interaction with them; or (2) approach any other partner due to preference for novelty. In both cases, we should have found that dogs approach the familiar partner significantly above or below chance level. However, dogs’ behaviour in the Test phase suggests that their lack of preference toward the specific partner might have been influenced by their level of motivation. In the absence of the balls, about 40% of the dogs did not approach any of the UMOs and 35% neither humans which suggests a lack of interest in the partners. Although most dogs approached at least one of the partners when balls were presented, subjects did not display attention to the partners here either.

To be able to discriminate (and choose) between a prosocial and antisocial partner, individuals also need to be able to discriminate between the partners’ based on their identity. Although Marshall-Pescini et al. (2011) reported that dogs prefer a generous donor over a selfish one in a food sharing situation, in a follow up study, Nitzschner et al. (2014) found that dogs relied only on the location of the humans and not their identity. Carballo et al. (2015) showed that in direct interaction when male vs female prosocial and antisocial partners were presented, dogs discriminated them after six trials, but in the case where both were female humans, more experience was required. In a recent study, they also found that in a problem solving task, dogs do not turn first to the prosocial human partner, although they displayed more gaze at this partner overall (Carballo et al. 2020). Thus, it seems that discriminating between humans can be difficult even when choice is made immediately after gaining experience with them. Although it should be noted that social evaluation may also play a role in these context. In our novel study, we also found that dogs do not display immediate preference to a UMO that helped them obtain an unreachable object over a novel UMO, despite that subjects were tested with the familiar vs novel UMO immediately after a problem solving task similar to the Training phases presented here (Capitain et al. 2022, submitted).

Despite the absence of IR of the specific partner, dogs remembered the 30-min interaction with the novel agent (UMO) in general, even after a month. Thus, these agents seem to be accepted by dogs as an interactive partner across social contexts, and after longer periods as well, and thus might be used in studies on memory more widely. The present experiment is the first using robotic agents to study IR and long-term memory in animals. Our results overall suggest that short experience is not sufficient even with human partners for individual identification, following a delay. Future studies should explore how much and what type of experiences are needed for IR of a social partner.

## Supplementary Information

Below is the link to the electronic supplementary material.

Supplementary file1 (DOCX 2555 KB)

Supplementary file2 (XLSX 68 KB)

Supplementary file3 (XLSX 36 KB)

Supplementary file4 (MP4 233880 KB)

## Data Availability

All data generated or analysed during this study are included in this published article and its supplementary information files.
