# Supplementary material for: Individual recognition and long-term memory of inanimate interactive agents and humans in dogs
Source: Anim Cogn. 2022 May 5;25(6):1427–42. doi: 10.1007/s10071-022-01624-6 (PMC9652224; doi:10.1007/s10071-022-01624-6)
Supplement: Supplementary file 1 — Supplementary file1 (DOCX 2555 KB) [file 10071_2022_1624_MOESM1_ESM.docx]

**Electronic supplementary material for:**

Individual recognition and long-term memory of inanimate interactive agents and humans in dogs
in *Animal Cognition*

Judit Abdai^1^, Dalma Bartus^2^, Sylvain Kraus^3^, Zsuzsanna Gedai^4^, Beatrix Laczi^4^, Ádám Miklósi^1,4*^

^1^MTA-ELTE Comparative Ethology Research Group, Budapest, Hungary

^2^University of Veterinary Medicine, Budapest, Hungary

^3^Paris 13 University, Paris, France

^4^Department of Ethology, Eötvös Loránd University, Budapest, Hungary

* Corresponding author: adam.miklosi@ttk.elte.hu

**Experiment 1**

**1. Additional information about the methods – Test partners and apparatus**

We only had one remote controlled base, thus when all UMOs were in the room at the same time (*Test phase*), we used identical hand-made bases (from wood and black wheels). With the embodiments on the bases, the UMOs appeared to be the same as the UMO with the original base (Fig. S1).


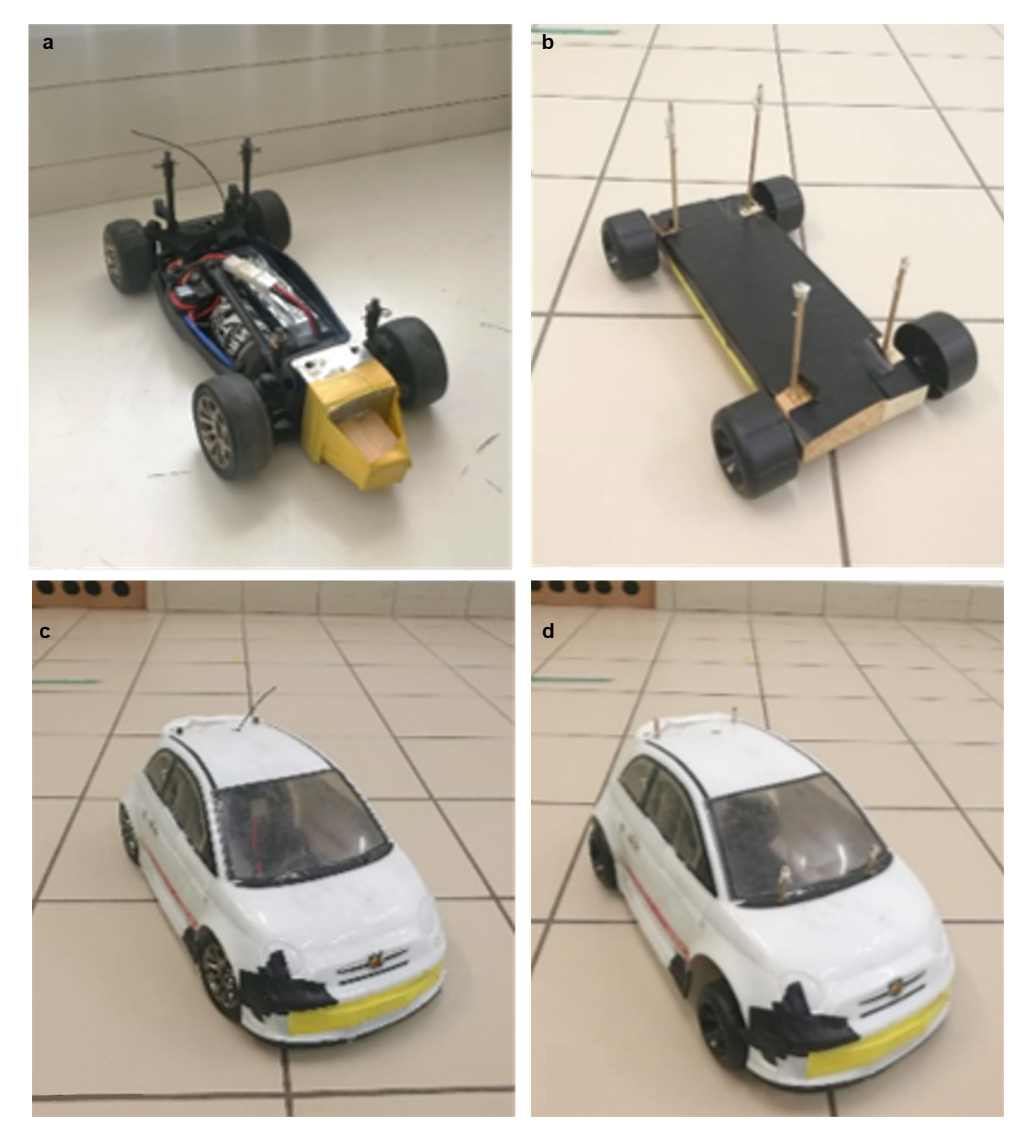


***Fig. S1*** *Real and hand-made bases of the UMO: (a) Remote-controlled base; (b) Hand-made base; (c) Remote-controlled base with the original plastic embodiment; (d) Hand-made base with the original plastic embodiment*

We used cartonplast sheets within the cage to facilitate the motion of the UMO. To prevent smaller dogs to go inside, we placed a wooden-cartonplast object close to the opening, inside the cage (barrier). The barrier had a metal sheet on one side, and magnets on the other side, thus the UMO could attach to the plate when the barrier was between them (Fig. S2b). To be able to push the ball more easily, we used a curved piece of metal (covered with duct tape to soften the edges) that could be attached to the front of the UMO (Fig. S2a). We placed the barrier and the curved metal in the room before the Training phase as well as the other equipment. After putting the ball inside the cage, E1 also placed the barrier inside the cage about 15 cm from the opening, in case the dog was small enough to fit in the cage.


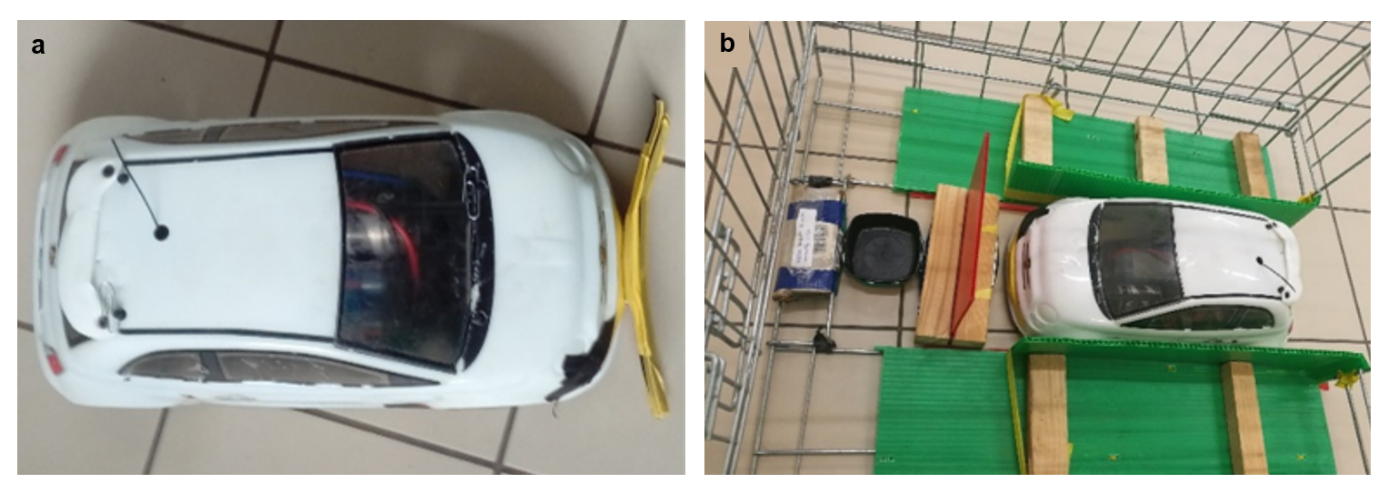


***Fig. S2*** *Objects used in the experiment: (a) UMO with the curved metal on its front; (b) The cartonplast sheets used to guide the UMO inside the cage, the wooden-cartonplast object used as a barrier, and the small black plate*

After the dog took away the ball brought out by the UMO, E2 took the plate off the UMO and attached the curved metal to its front. At the beginning of the following trial, E1 took the curved metal off the UMO after entering, and took the ball from the dog/E2. Throughout the test, the owner and the dog used Door A, and E1 used door B (see Fig. 3 in the main text). E2 was the same person throughout the experiment (JA), but E1 was either one of two persons (SK and DB); E1 was the same person in the Familiarization and Retest sessions of the same dog.

**2. Questionnaire filled out by owners after the test**

After the Retest session, we sent a questionnaire to owners via email, which contained questions regarding the dog’s general playing habits (Table S1).

***Table S1*** *Questionnaire filled out by owners after the Retest session*

| **Question** | **Response** |
| --- | --- |
| Dog’s name | *Short response* |
| Breed | *Choice from a list* |
| What type of ball does your dog like to play with? | - Any type - Only with some specific type - Only with its own ball |
| How your dog does likes to play with ball? | - Run after the ball and then chewing on it - Runs after it and brings it back |
| If your dog likes to run after a ball: Does it matter how well you throw the ball? | Scale from 1 to 5 |
|  | ➀ No, the dog is happy even with a slight movement  ➄ I always need to throw the ball to a long distance |
| If your dog likes to chew a ball: How difficult it is to take it away | Scale from 1 to 5 |
|  | ➀ The dog is never willing to give it  ➄ The dog gives the ball to me very easily |
| Does your dog put the ball down in front of you without a command? | Scale from 1 to 5 |
|  | ➀ I have to give a command/take it out of his/her mouth  ➄ The dog always puts down the ball without asking |
| If your dog does not give the ball by him/herself: Is it enough to give a command or you need to take it away? | Scale from 1 to 5 |
|  | ➀ I have to take it away from his/her mouth  ➄ The dog always give it when I give the command (up to ca. 3 commands) |
| Does your dog like to play with a ball with other dogs? | Scale from 1 to 5 |
|  | ➀ Never  ➄ Frequently |
| Does your dog play with other dogs in any other way? | Scale from 1 to 5 |
|  | ➀ Never  ➄ Frequently |
| Does your dog share its toys with other dogs? | Scale from 1 to 5 |
|  | ➀ Never  ➄ Frequently |
| Is your dog tend to take the ball away from other dogs? | Scale from 1 to 5 |
|  | ➀ Never  ➄ Frequently |
| Is your dog tend to release/leave alone a ball that belongs to another dog (the other dog plays/played with it)? | Scale from 1 to 5 |
|  | ➀ Never  ➄ Frequently |
| Is your dog tend to release/leave alone a ball that was touched by another dog? | Scale from 1 to 5 |
|  | ➀ Never  ➄ Frequently |
| Is your dog willing to give a ball to an unfamiliar person? | - *Yes*, but only if he/she gives a command to release (the person cannot take it away from the mouth) - *Yes*, but only by taking it away from the dog's mouth (does not give it upon command) - *Yes*, the unfamiliar person can ask and take the ball away as well - *No*, the dog does not give it to an unfamiliar person |

**3. Inter-coder reliabilities**

Inter-coder reliability was acceptable for latency of the first approach of the UMO in both Test trials (Spearman’s rho: Test Trial 1, ρ = 0.891, *p* = 0.001; Test trial 2, ρ = 0.996, *p* < 0.001). To test inter-coder reliability of the looking at the UMOs variable (within-trial dynamics of look) in the Test phase, we exported the specific sections of the coding sheets of both coders from the Solomon Coder and checked the correspondence between coders for all data points (overall ca. 300 frames per subject). Inter-coder reliability was tested calculating Cohen’s kappas. Result indicated acceptable agreement between coders (mean *± SD* Cohen’s kappa: κ = 0.621 *± 0.12*). Inter-coder reliability was also acceptable for latency of first look at the UMO in the Training phases (Spearman’s rho: ρ = 0.635, *p* < 0.001), and also for putting the ball down for the UMO or to a human (Spearman’s rho: UMO, ρ = 0.704, *p* < 0.001; Human, ρ = 0.643, *p* < 0.001).

**4. Analysis of the playing behaviour of dogs**

We analysed whether dogs were more likely to put the ball down to the UMO or to one of the humans in the room (owner and E2), by using binomial Generalized Linear Mixed Model (binomial GLMM) (“lme4” package). Some dogs in general play with the ball in an interaction with a human, but others prefer to chew the ball alone; the latter group of dogs usually do not give the ball even to a human (their owner) readily. Thus, first we analysed whether dogs’ tendency to put the ball down for the UMO or a human depended on their preferred playing style (indicated by the owner in the questionnaire). Following this, we tested whether the time elapsed between the two occasions had an effect on dogs’ behaviour (group), and whether dogs were more likely to put the ball down to the UMO or a human in either sessions (session).

We did not find an interaction between dogs’ playing style and putting the ball down to the UMO or a human (binomial GLMM, $\chi_{1}^{2}$ = 2.908, *p* = 0.088). However, overall dogs were more likely to put the ball down to the owner or E2, than to the UMO ($\chi_{1}^{2}$ = 39.469, *p* < 0.001; UMO vs human: β ± SE = -0.840 ± 0.135; *p* < 0.001). Dogs’ general playing style also had an effect on whether dogs put the ball down in our test ($\chi_{1}^{2}$ = 7.782, *p* = 0.005) (Fig. S3). Dogs that prefer to play in interaction with their owner (based on the owner’s report) were more likely to put the ball down for the UMO or any of the humans, than dogs that prefer solitary play (Interaction vs Alone: β ± SE = 1.260 ± 0.435; *p* = 0.004). This indicates that the *putting the ball down* behaviour in our study reflects the general preference of dogs, irrespectively of the specificity of the study. Thus further analyses regarding this behaviour were tested only in case of dogs that prefer to play in interaction in general (N = 23).


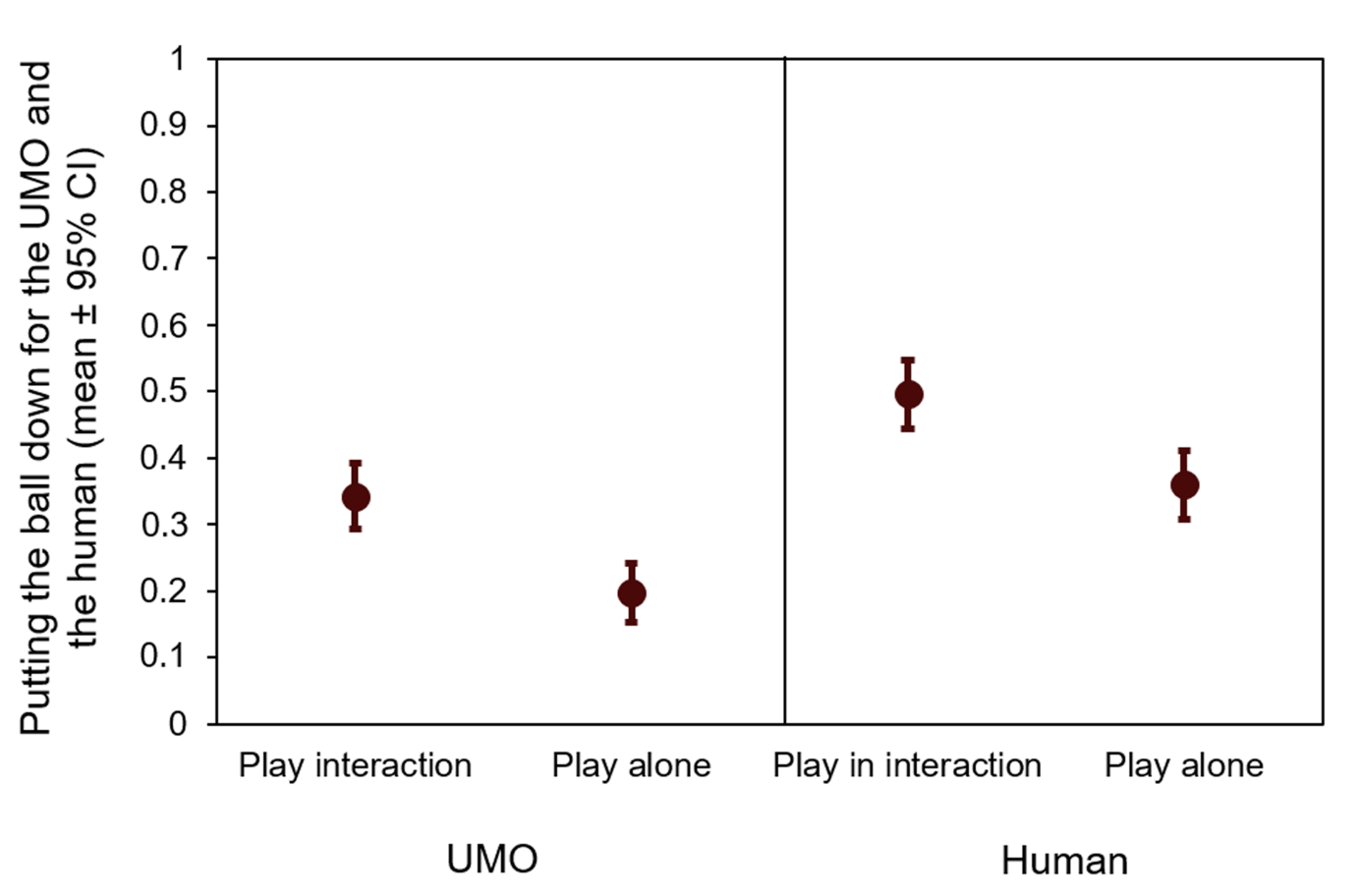


***Fig. S3*** *Putting the ball down for the UMO and for a human, depending on the preferred playing style of the dog*

The interaction of group and session had no effect on whether dogs put the ball down for the UMO or to any of the humans (binomial GLMM, LRT: Group x Session, $\chi_{2}^{2}$ = 0.216, *p* = 0.898). The time elapsed between the two sessions did not have a main effect (Group, $\chi_{2}^{2}$ = 0.379, *p* = 0.827). Dogs showed similar behaviour during the Familiarization and Recognition sessions, regarding their tendency to put down the ball in front of the UMO or a human (Session, $\chi_{2}^{2}$ = 2.694, *p* = 0.101).

We carried out further analysis regarding dogs’ playing behaviour with the UMO. We compared dogs’ playing behaviour reported by the owner to whether they put the ball down to the UMO. Considering that owners rated dogs on a scale from 1 to 5 in all cases, instead of using the original data set of dogs with the binary variable, we assigned them a score between 1 and 5 based on the percentage of trials the dog put the ball down to the UMO: number of the trials the dog put the ball down to the UMO divided by the number of trials of the specific dog, multiplied by 100 (Table S2). We applied Cohen’s kappa (“irr” package) to compare these scores with how likely the dog is (1) to play with a ball with other dogs; (2) to play with other dogs in any other way; (3) to share its toys with other dogs; (4) to take the ball away from other dogs; (5) to release/leave alone a ball with which another dog was playing previously; (6) to release/leave alone a ball that was touched by another dog; and (6) whether dogs’ score of putting the ball down to the UMO reflects whether their behaviour in a playing interaction depends on how well a human throws the ball. Two dogs were excluded from this analysis, because their owners did not fill out the questionnaire.

***Table S2*** *The specific scores assigned based on the percentage of trials in which the dog put the ball down for the UMO*

| **Percentage** | **Score** |
| --- | --- |
| 0-19 | 1 |
| 20-39 | 2 |
| 40-59 | 3 |
| 60-79 | 4 |
| 80-100 | 5 |

We did not find any relation between dogs’ general behaviour in a playing situation, and their likeliness to put the ball down to the UMO (Cohen’s kappa: playing ball with other dog, κ = -0.051; playing with dogs without ball, κ = -0.040; sharing their ball with other dogs, κ = 0.052; take away: κ = -0.012; release/leave alone a ball with which another dog was playing before, κ = 0.049; release/leave alone a ball that was touched by another dog, κ = -0.004; quality of throwing the ball, κ = -0.161).

**Experiment 2**

**5. Inter-coder reliabilities**

Inter-coder reliability was acceptable for latency of first approach of the UMO in both Test trials (Spearman’s rho: Test Trial 1, ρ = 0.986, *p* < 0.001; Test trial 2, ρ = 1.000, *p* < 0.001). To test inter-coder reliability of the looking at the UMOs variable (within-trial dynamics of look) in the Test phase, we exported the specific sections of the coding sheets of both coders from the Solomon Coder and checked the correspondence between coders for all data points (overall ca. 300 frames per subject). Inter-coder reliability was tested calculating Cohen’s kappas. Result indicated acceptable agreement between coders (mean *± SD* Cohen’s kappa: κ = 0.697 *± 0.09*). Inter-coder reliability was also acceptable for latency of first look at the UMO in the Training phases (Spearman’s rho: ρ = 0.922, *p* < 0.001).

However, although the correlation was significant for putting the ball down for the partner, and to the owner assessed by the two coders, Spearman’s rho indicated moderate (Partner, ρ = 0.501, *p* < 0.001) and weak correlation only (Owner, ρ = 0.384, *p* < 0.001). Thus, we did not analyse this behaviour further.
